# Molecular Mechanism of Quorum-Sensing in *Enterococcus* *faecalis*: Its Role in Virulence and Therapeutic Approaches

**DOI:** 10.3390/ijms18050960

**Published:** 2017-05-03

**Authors:** Liaqat Ali, Mohsan Ullah Goraya, Yasir Arafat, Muhammad Ajmal, Ji-Long Chen, Daojin Yu

**Affiliations:** 1 College of Animal Sciences, Fujian Agriculture and Forestry University, Fuzhou 350002, China; liaqatpaksw@yahoo.com (L.A.); goraya_uaf@yahoo.com (M.U.G.); chenjl@im.ac.cn (J.-L.C.); 2 College of Life Sciences, Fujian Agriculture and Forestry University, Fuzhou 350002, China; arafat_pep@yahoo.com; 3 Department of Biosciences, Faculty of Science, COMSATS Institute of Information Technology, Islamabad 45550, Pakistan; muhammad.ajmal@comsats.edu.pk; 4 CAS Key Laboratory of Pathogenic Microbiology and Immunology, Institute of Microbiology, Chinese Academy of Sciences (CAS), Beijing 100101, China

**Keywords:** cytolysin, *Enterococcus faecalis*, Fsr, multidrug-resistant pathogen, quorum-sensing, quorum-sensing inhibitor, virulence factor

## Abstract

Quorum-sensing systems control major virulence determinants in *Enterococcus* *faecalis*, which causes nosocomial infections. The *E*. *faecalis* quorum-sensing systems include several virulence factors that are regulated by the *cytolysin* operon, which encodes the cytolysin toxin. In addition, the *E*. *faecalis* Fsr regulator system controls the expression of gelatinase, serine protease, and enterocin O16. The cytolysin and Fsr virulence factor systems are linked to enterococcal diseases that affect the health of humans and other host models. Therefore, there is substantial interest in understanding and targeting these regulatory pathways to develop novel therapies for enterococcal infection control. Quorum-sensing inhibitors could be potential therapeutic agents for attenuating the pathogenic effects of *E*. *faecalis*. Here, we discuss the regulation of cytolysin, the LuxS system, and the Fsr system, their role in *E*. *faecalis*-mediated infections, and possible therapeutic approaches to prevent *E*. *faecalis* infection.

## 1. Introduction

*Enterococcus faecalis* is an aerotolerant, Gram-positive bacteria that is distributed widely in the natural environment, and in the gastrointestinal tracts of humans, animals, and insects. Among different enterococcal species, *E*. *faecalis* causes urinary tract infections, bacteremia, prosthetic joint infection, abdominal-pelvic infections, and endocarditis [1,2]. The most important features of *E*. *faecalis* are their high adaptability under harsh environmental conditions and their potential development of antibiotic resistance [3,4,5].

Antibiotic treatment eliminates vulnerable bacteria from the bacterial population, leaving resistant bacteria to grow and multiply. In *E*. *faecalis*, acquired elements, including antibiotic resistance genes, are estimated to represent over 25% of its genome [6]. Acquired and intrinsic resistance mean that *E*. *faecalis* shows resistance to a variety of antibiotics [4]. Virulence-specific therapeutics could avoid the selective pressure posed by antibiotics. Therefore, alternative anti-virulence therapeutic strategies, such as inhibition of quorum-sensing systems, could be sought to target this opportunistic pathogen.

The quorum-sensing system is the population density-dependent regulatory mechanism by which bacteria communicate via signaling molecules, called autoinducers [7]. Generally, in quorum-sensing, bacteria produce autoinducers, and these molecules accumulate in the environment with the increase in the cell density. The role of these autoinducers depends on the location of their receptors, which are present on cell surface or in the cytoplasm. An autoinducer activates its cognate receptor specifically, which then activates the transcription of quorum-sensing genes [8]. This phenomenon provides a mechanism for bacteria to synchronize their social behavior, to communicate among themselves, and to regulate gene expression in response to their population density. Gram-positive bacteria contain another type of quorum-sensing, in which autoinducers are transported back into the bacterial cytoplasm, where they interact with a specific transcription factor, thereby regulating gene expression [8].

With the advent of proteomic studies in bacteria, it is revealed that the quorum-sensing system not only regulates their specific regulon but also control the expression of many other proteins ranging from surface proteins, transcription factors, virulence, biofilm formation to metabolic pathways [9,10,11]. Proteomics allow a comprehensive understanding of quorum-sensing phenomena and make it possible to better discern patterns of proteins expression in bacteria. It is estimated that over 23% of bacterial proteome can be regulated by quorum signaling [12]. Shao et al. have used the proteomic technique of 2D-PAGE (two-dimensional polyacrylamide gel electrophoresis) and identified 15 differentially expressed quorum-sensing-dependent proteins in vancomycin-resistant *E*. *faecalis* [10]. Following its identification, quorum-sensing plays a basic role in biofilm development. Using a proteomic approach (2D-PAGE coupled with mass spectrometry), Piras and coworkers reported that quorum-sensing-related LuxS enzyme (also known as *S*-ribosylhomocysteine lyase) is highly secreted in multidrug-resistant bacteria [9]. These results demonstrate that multidrug-resistant phenotype can also participate in a variety of regulatory and metabolic functions at high cell densities as well as an increase in their respective autoinducer concentrations. Extensive studies are still required to explore the detailed interaction of quorum-sensing proteins and whole proteomic expressions.

In Gram-positive bacteria, autoinducers are modified oligopeptides that are exported out of the cell. Outside the cell, because of their higher concentrations, different autoinducers bind with membrane-bound histidine kinase receptors. Histidine kinase is initially autophosphorylated and then activates the response regulator, which in turn activates the quorum-sensing regulon [13]. Peptide autoinducers are used commonly by Gram-positive bacteria, and acyl-homoserine lactone is produced by Gram-negative bacteria. By contrast, autoinducer 2 (AI-2) molecules are used for intra- and inter-species communication in Gram-positive and negative bacteria [11,14]. Currently, the “SigMol” repository database contains 182 unique autoinducer molecules identified inprokaryotes [15].

In *E*. *faecalis*, there are several sex pheromone-responsive plasmids that encode bacteriocins, aggregation substances, and a broad range of antibiotic resistance determinants (reviewed in [16]). Some of them, for example pCF10, pAD1, pPD1, pOB1, and pAM373, encode pheromones (cCF10, cAD1, cPD1, cOB1, and cAM373, respectively). The pheromone peptides (autoinducers) are transported through the ATP-binding cassette transport system [17]. Accumulation of these autoinducers in the extracellular milieu is sensed by their corresponding recipients to regulate conjugation-related mating functions [18]. Furthermore, once a recipient acquires a plasmid, the donor cells secrete the pheromone inhibitor. These inhibitors then downregulate the conjugation genes in donor cells, which controls the transfer of genes throughout a population [19]. *E*. *faecalis* also uses a cyclic peptide molecule, known as gelatinase biosynthesis-activating pheromone (GBAP), and CylL_S_, as autoinducers, which interact with their transmembrane receptors FsrC, and CylR1, respectively, during the quorum-sensing process [20,21].

Understanding the mechanisms by which *E*. *faecalis* populations regulate their mutual behavior, and how these behaviors are linked with the switch between commensal and pathogenic states, deserve attention. The role of *E*. *faecalis* system regulator (Fsr), cytolysin, and LuxS quorum-sensing regulatory systems, and possible therapeutics approaches, are discussed in this review. The Fsr and cytolysin regulatory systems in *E*. *faecalis* regulate much of their pathogenicity and have been documented in several studies [22,23,24,25,26], while the role of the LuxS regulatory mechanism in *E*. *faecalis* is less certain [10,27].

## 2. Fsr Mediated Quorum-Sensing

**Figure 1:**
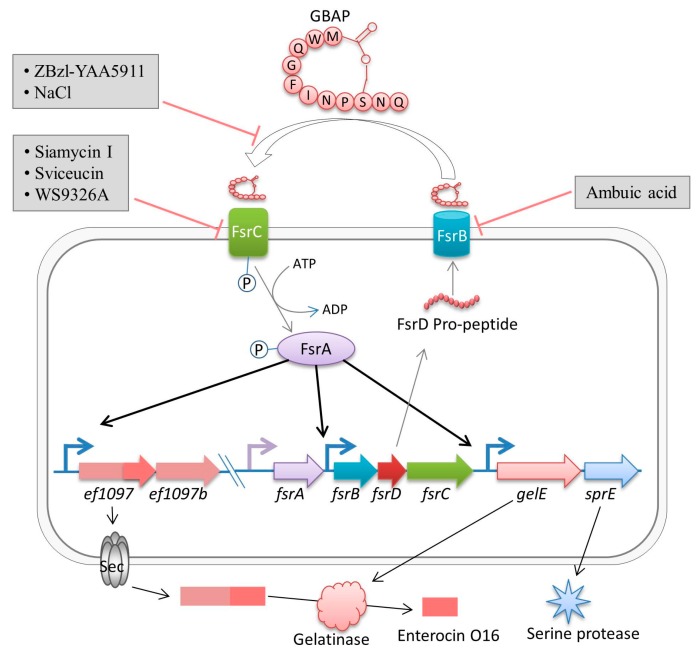
The Fsr quorum-sensing system and its regulation in *E*. *faecalis*. The FsrD propeptide (encoded by *fsrD*) is exported and processed to produce small lactone gelatinase biosynthesis-activating pheromone (GBAP) via FsrB. FsrC is part of a two-component regulatory system that responds to extracellular GBAP and phosphorylates the intracellular response regulator, FsrA, which then induces the expression of *ef1097*, *ef1097b*, *fsr* locus, *gelE* (encoding a gelatinase), and *sprE* (encoding a serine protease). The pre-proprotein (170 amino acids) encoded by *ef1097* is cleaved (N-terminal 34 amino acids are removed) and transported through the Sec-dependent pathway, where gelatinase further trims the precursor to form enterocin O16 (68 C-terminal amino acids). ZBzl-YAA5911 (competitively) and NaCl (concentration-dependently) inhibit the interaction of GBAP with FsrC. Ambuic acid inhibits FsrB activity. Siamycin I, Sviceucin, and WS9326A inhibit the phosphorylation of FsrC.

The *fsr* locus of *E*. *faecalis* encodes a two-component regulatory system that senses the cell density and regulates virulence [25,28]. The *fsr* locus is 2.8 kb in size and comprises four genes: *fsrA*, *fsrB*, *fsrD*, and *fsrC* [29]. Four genes, *gelE*, *sprE*, *ef1097*, and *ef1097b*, are directly dependent on the Fsr system [30]. The *fsrA* gene encodes the FsrA protein, which belongs to the LytTR family of DNA-binding domains [31]. The binding of phosphorylated FsrA to LytTR-binding sites in the upstream region of *ef1097*, *fsrB*, and *gelE* suggested that FsrA is a response regulator of the Fsr system [31]. Notably, *fsrA* transcription is under the control of a constitutive promoter; therefore, it is independent of the Fsr quorum-sensing system [32]. The *fsrB* gene encodes a transmembrane protein, FsrB, which belongs to the accessory gene regulator protein B (AgrB) family. FsrB processes a propeptide, FsrD (encoded by *fsrD*), to generate GBAP (a lactone ring containing a short cyclic peptide of 11 amino acid residues), which is further exported out of the cell [29] (Figure 1). The fourth gene, *fsrC*, encodes the transmembrane histidine protein kinase FsrC, the sensor-transmitter of the *fsr* operon [20].

The *fsr* operon encodes a *Staphylococcus aureus* Agr (accessory gene regulator)-like system, which is a quorum-sensing system responsible for the regulation of virulence in the host tissues [13]. With the growth of an *E*. *faecalis* population, the concentration of GBAP rises in the external environment. Accumulation of GBAP in the extracellular milieu is sensed by FsrC, which is then phosphorylated to activate the response regulator FsrA [20]. Thus, this two-component regulatory system, consisting of FsrC (sensor) and FsrA (response regulator), responds to the accumulated GBAP, and is mainly involved in cell-cell communication in *E*. *faecalis*. Phosphorylated FsrA then regulates the transcription of *fsrBCD*, *gelE*-*sprE* operons, and *ef1097* locus (*ef1097* is located 800 kb upstream of the *fsr* operon) [31]. Mutation of *fsrABC* affected the downstream transcriptional response of *gelE* and *sprE* [25]. Therefore, *fsrABC* are essential for the regulatory functions of the *fsr* regulon [32]. Deletion of *fsrA*, *fsrB*, or *fsrC* abolished the expressions of *gelE* and *sprE* completely; however, both were highly expressed in wild-type *E. faecalis* strain OG1RF [32]. Similarly, the expression of *ef1097* was upregulated by 214-fold in the late log phase and by 10-fold in early stationary phase in *E. faecalis* OG1RF compared with the *fsrB* mutant strain [33]. Interestingly, Dundar et al. identified enterocin O16 (also known as EntV and enterococcin V583), which is encoded by *EntV* gene (the *ef1097* locus) [34,35,36]. The pre-proprotein encoded by *ef1097* is translocated by the Sec system, and is further modified by an extracellular gelatinase [34]. Moreover, Sec system-mediated translocation and further modification of the large precursor pre-proprotein by gelatinase make it unclear whether or not enterocin O16 is a regular bacteriocin-type peptide. Nevertheless, *E*. *faecalis* is intrinsically resistant to enterocin O16, which indicates that the Fsr system also has antimicrobial and antifungal activity at higher cell densities [34,35]. However, additional studies are needed to investigate the structure and function of enterocin O16 in *E*. *faecalis*, and how the self-protective mechanism operates to protect *E*. *faecalis* from the harmful effects of enterocin O16. In addition to *gelE*, *sprE*, and *ef1097*, several genes are regulated indirectly by the Fsr system that have roles in surface adhesion, autolysis, and biofilm development [30,33]. However, the greater variability of genes regulated by *fsr* suggests that this system is not only involved in virulence, but also alterations to metabolic activities, and biofilm-related components could play an important role.

### Pathogenesis of Fsr Mediated Quorum-Sensing

Gelatinase and serine protease, encoded by *gelE* and *sprE*, respectively, are regulated positively by the Fsr quorum-sensing system [30,32,37]. Both *gelE* and *sprE* are located adjacent to the *fsr* genes, and are regulated by a common promoter [32] (Figure 1). Collectively, these proteases, and the Fsr quorum-sensing system, contribute to virulence, the degradation of host tissues, and biofilm formation [38,39,40,41,42,43].

The *gelE* gene encodes gelatinase, which is a mature polypeptide of 318 amino acid residues, with a molecular mass of about 34.5 kDa [44]; 14 C-terminal amino acid residues are removed during processing to fully activate the protease activity of gelatinase [45]. Gelatinase has been classified as a metalloprotease II, capable of hydrolyzing gelatin; collagen; fibrin; fibrinogen; hemoglobin; complement components C3, C3a, and C5a; endothelin-1; casein; and other small peptides [44,46,47,48], which suggests its possible role in *E*. *faecalis* pathogenesis. Gelatinase activates the autolysin that is responsible for biofilm formation [43]. It also promotes *E. faecalis* in vitro translocation into the T84 human carcinoma cell line [49]. Moreover, gelatinase activates the protease-activated receptor-2 (PAR-2), which induced chronic intestinal inflammation in mice [50,51]. PAR-2 expression and serine protease activity have been observed in dogs with inflammatory bowel disease [52], which suggested that this receptor might be a risk factor for intestinal diseases mediated by the Fsr quorum-sensing system.

Among clinical *E*. *faecalis* isolates, the *gelE* gene was detectable in more than 78% of the isolates from urine, wounds, the genital tract, and blood [53]. Other studies noted that phenotypic gelatinase activity ranged from 13% to 70% of oral cavity isolates, which suggested their possible virulent role in the hospital environment [54,55]. In addition, indications of gelatinase-related virulence are also observed commonly in animals and insects [30,37,56] (Table 1).

The *sprE* gene encodes a serine protease that has been characterized as a glutamyl endopeptidase I, with a mass of 25 kDa, and is a virulence factor of *E. faecalis* [25,65]. This protease contributes to pathogenesis in a variety of models, including animals [25,62,66], plants [64], and insects [30,39,41]. However, the exact mechanism of virulence in these hosts is poorly understood.

The role of the Fsr quorum-sensing system in virulence in vivo has been assessed in several experimental host models, including mice, rabbits, *Caenorhabditis *elegans**, *Arabidopsis thaliana*, and *Drosophila melanogaster* (Table 1). In these experimental models, the activities of Fsr quorum-sensing were examined either by mutation (disruption or deletion in *fsrA*, *fsrB*, or *fsrC*) or interference with GBAP-mediated quorum-sensing by various treatments. In 1998, Singh and coworkers used isogenic strains to evaluate the virulence effect of gelatinase in mice [59]. Their results indicated that inoculation (8 × 10^8^ CFU/mL) of *gelE*-defective mutant strain delayed mortality significantly compared with *E. faecalis* OG1RF. To further examine the delayed mortality, Qin et al. constructed insertion mutants of *fsrA*, *fsrB*, and *fsrC*, and downstream gene *sprE*, to test the role of the Fsr quorum-sensing in virulence, using a mouse peritonitis model [25]. Based on their observations, *gelE* and *sprE* are Fsr quorum-sensing-mediated virulence factors in mouse peritonitis. Similarly, double mutants (*gelE* and *sprE*) in the *fsrA*, *fsrB*, and *fsrC* mutant background showed more attenuated virulence compared with that of the *gelE* insertion mutant in a *C. elegans* infection model [41]. Moreover, double mutants (*gelE* and *sprE*) or triple mutants (*fsrB*, *gelE* and *sprE*) displayed a more attenuated phenotype than the *fsrB* mutant in a *D*. *melanogaster* model [30]. Collectively, these results indicated that, in addition to *gelE* and *sprE*, the Fsr quorum-sensing system, at a high cell density, might be regulated other virulence factors that play a significant role in the in vivo infection of *E. faecalis.*

Transcriptome analyses revealed that in addition to *gelE*, *sprE*, and *ef1097*, approximately 75 other genes are deregulated by ≥5-fold by the Fsr system, including those related to surface proteins (EbpR), biofilm formation (BopD), and metabolic pathways [33]. In another study, Teixeira et al. found that the external addition of GBAP to *fsrB* mutant strains upregulated the *lrgAB* genes [30]. These genes are responsible for murein hydrolase activity and are regulated by the *lytSR* operon in biofilm formation and extracellular DNA release [67]. In addition, gelatinase and serine protease are also involved in the regulation of an autolysin, *N*-acetylglucosaminidase (AtlA), which has a role in the fratricide and extracellular DNA release during biofilm formation [43]. Collectively, these studies suggested that bacteria with a deletion of *gelE* exhibited a tendency toward autolysis. By contrast, the deletion of *sprE* increased the fratricide activity, which suggested that the serine protease inhibited lysis of the cells [68]. Both gelatinase and serine protease control cell lysis by interacting with and changing the binding capacity of AtlA to the cell wall [43]. Moreover, gelatinase also cleaves a collagen adhesion protein (Ace, a bacterial protein), which affects *E*. *faecalis*’s ability to adhere to collagen fibers. The activity of Ace, mediated by gelatinase, suggests a common role of the Fsr system in colonization or dissemination [69]. The Fsr quorum-sensing system and glycosyl transferases (GTFs, encoded by *epaI* and *epaOX*) promote biofilm formation [38]. GTFs might be involved in the synthesis and processing of cell wall polysaccharides, which sequester the antibiotics present in the vicinity of cell walls, thus prevent absorption of the antibiotics [38]. However, further studies are required to understand the exact mechanisms of Fsr’s functions. Based on the studies mentioned above, it is speculated that there might be additional systems that play different roles in *E*. *faecalis* virulence, either directly or indirectly; however, they have yet to be identified. Understanding Fsr quorum-sensing would help to develop new and effective antivirulence drugs against *E*. *faecalis* pathogenesis.

## 3. Cytolysin Regulation

*E*. *faecalis* cytolysin is a pore-forming, Type-A lantibiotic [70], which has been classified more recently as a member of the two-component Class I lantibiotic enterocins (the bacteriocins produced by enterococci), which are post-translationally modified [71,72]. These two-component linear precursor peptides are encoded by two genes, *cylL_L_* and *cylL_S_*, which are located in the *cytolysin* operon [23,73]. When the CylL_S_ concentration increases to a threshold level, it promotes the autoinduction of the *cytolysin* operon by a quorum-sensing mechanism [74]. The resulting toxicity of cytolysin has been observed in a wide range of organisms, including eukaryotes and prokaryotes [5,24,26,39,74,75,76,77].

**Figure 2:**
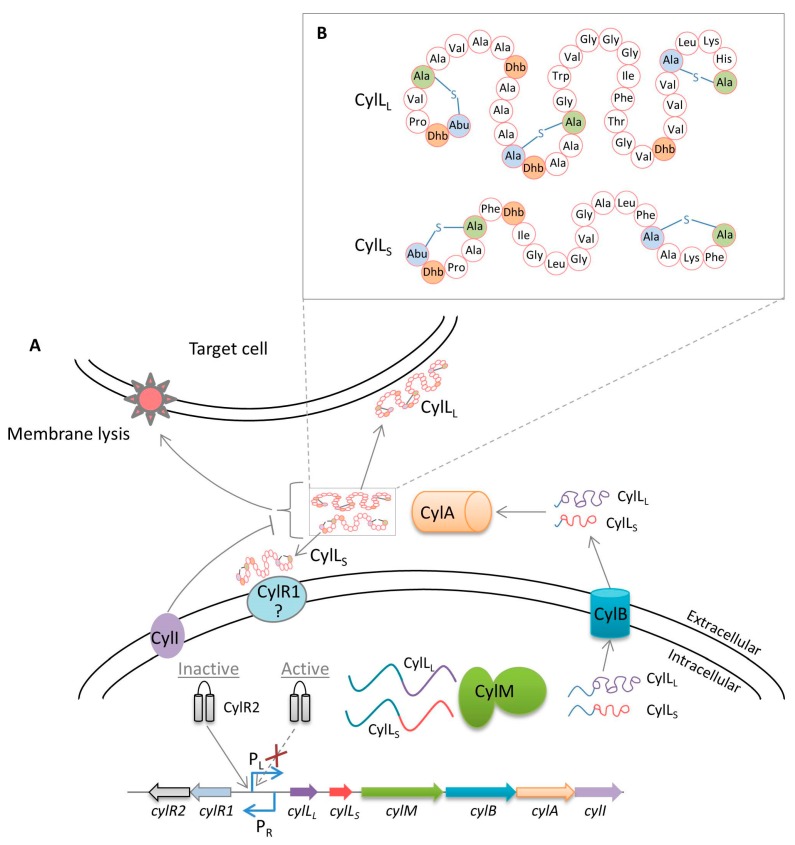
The cytolysin quorum-sensing system in *E*. *faecalis*. (**A**) The toxin structural components CylL_L_ (larger subunit) and CylL_S_ (smaller subunit) are encoded by *cylL_L_* and *cylL_S_*. CylL_L_ and CylL_S_ core peptides are post-translationally modified by CylM, after which these peptides are further processed and transported by CylB. Extracellular protease, CylA, removes six amino acid residues (leader peptide) from both CylL_L_ and CylL_S,_ making them active toxin subunits. The signal transduction mechanism linking the binding of extracellular CylL_S_ to CylR1 at the membrane with the dissociation of CylR2 from the P_L_ promoter is currently unknown. CylI provides self-immunity against cytolysin; (**B**) Structures of the CylL_L_ and CylL_S_ mature cytolysin subunits [82].

The *cytolysin* operon is located on the bacterial chromosome within the pathogenicity island, or on the pAD1 plasmid, and comprises eight genes [21,73,78] (Figure 2A). Two genes, *cylR1* and *cylR2*, encode regulatory proteins, and are transcribed by a separate promoter (P_R_) and are thus transcribed divergently [21]. The remaining six genes (*cylL_L_*, *cylL_S_*, *cylM*, *cylB*, *cylA*, and *cylI*) encode structural and functional proteins. Two structural genes, *cylL_L_* and *cylL_S_*, encode a large subunit of 68 amino acids (a 30-residue leader peptide at the N-terminus and a 38-residue core peptide at the C-terminus) and a small subunit of 63 amino acids (a 42-residue leader peptide at the N-terminus and a 21-residue core peptide at the C-terminus), respectively [75]. The products of the *cylL_L_* and *cylL_S_* genes are post-translationally modified by lanthionine synthetase (CylM). CylM dehydrates Thr and Ser residues in the core peptide to generate (*Z*)-2,3-didehydrobutyrine (Dhb) and 2,3-didehydroalanine (Dha), respectively [79]. The nucleophilic Michael-type addition reaction is then initiated by cysteine residues, resulting in the formation of thioether bonds between Dhb or Dha and nucleophilic cysteine side chains. Finally, this generates modified peptides containing methyllanthionine (starting from dehydrated Thr) or lanthionine (starting from dehydrated Ser) structures, which are both unusual amino acids in the peptides [77]. Intriguingly, Tang and van der Donk showed that one lanthionine bridge in CylL_S_ and two in CylL_L_ exhibit an LL configuration, which is a uniquely different stereochemistry compared with previously characterized lantibiotics [80]. In addition, formation of the LL stereochemistry is substrate (CylL_L_ and CylL_S_)-dependent, but not CylM-dependent [81]. CylM contains a dehydration domain that is involved in the hydrolysis of N-terminal amino acid residues of CylL_S_ (three Thr and one Ser) to three Dhb and one Dha, respectively. In addition, the CylM C-terminal cyclization domain is thought to be responsible for the thioether crosslinks [82] (Figure 2B). Furthermore, CylB removes most of the CylL_L_ and CylL_S_ leader peptides and then transports them into the extracellular environment via an ATP-binding cassette transporter [83]. The *cylA* gene encodes a serine protease, CylA, which removes six amino acids (Gly-Asp-Val-Gln-Ala-Glu) from the N-terminus of the secreted toxin subunits, thus converting them to active toxin subunits on the cell surface [21,75]. However, these subunits form an inactive oligomeric complex in the presence of host cells; CylL_L_ exhibits preferential binding to the host cell membrane, which allows free CylL_S_ to become signaling molecules [74]. Free CylL_S_ acts as an autoinducer, and at a certain threshold concentration, binds to the membrane-bound CylR1. This disrupts the interaction of the repressor protein (CylR2) with the operator sequence, which further leads to the expression of the *cytolysin* operon [21,84]. However, CylR1 signal transmission from extracellular CylL_S_ to intracellular CylR2 has not been completely determined. The final gene, *cylI*, encoding immunity protein CylI, is located immediately adjacent and downstream to the *cylA* [85]. In addition, CylI has been predicted to harbor a putative zinc binding metalloprotease motif and transmembrane domains [77]; however, the precise protective mechanism against the cytolysin toxicity involving CylI has yet to be determined.

### Virulence of Cytolysin 

About 30% of the *E. faecalis* strains and 60% of clinical isolates are cytolysin producers [3,72]. The toxicity of *E. faecalis* cytolysin has been evaluated in vivo, in vitro, and for its clinical outcomes (Table 1). Cytolysin is active against a wide variety of Gram-positive bacteria, including clostridia, lactobacillales, and staphylococci [86,87]. The exact mode of action of cytolysin is unclear; however, its subunits, CylL_L_ and CylL_S_, bear some similarity to the well-studied bacteriocin lacticin 3147 [88], which is a pore-forming two-component (lacticin A1 and lacticin A2) lantibiotic, produced by *Lactococcus lactis* [89]. Pore-formation by lacticin 3147 takes place in three steps. First, the lacticin A1 subunit is associated with the membrane and lipid II. Secondly, its interactions with lacticin A2 form a high-affinity three-component complex. Finally, the C-terminus of lacticin A2 in the complex is translocated into the membrane to form a pore [90]. Whether the subunits of cytolysin interact in the same way, as well as their mechanism of action and spectrum of activity, has yet to be identified.

The cytolysin toxin can cause lysis of the human erythrocytes, polymorphonuclear leukocytes, retinal cells, and intestinal epithelial cells [23,26,91], which suggested its causative role in hemolysis, endophthalmitis, compromised immunity, and intestinal diseases. The virulent behavior of cytolysin was also evident from a study in which mice were given intraperitoneal injection with strains harboring the *cytolysin* operon and isogenic non-cytolysin strains [92]. The cytolysin phenotype was observed to be potentially more virulent than the isogenic non-cytolysin strain in mice [92]. However, another study showed that an inoculum containing Sterile Rat Fecal Extracts (SREF) enhanced the severity of peritonitis in mice [59]. A retrospective study revealed that the cytolytic strains in enterococcal bacteremic infection resulted in a five-fold increase in the risk of mortality compared with the patients infected with non-cytolytic *E*. *faecalis* strains [93]. Bacteremia isolates have also been shown to have significantly higher levels of cytolysin than the endocarditis strains and strains in healthy individuals [94].

La Rosa et al. established a method of real-time monitoring of *gelE*- and *cytolysin*-promoter activity in *Galleria mellonella* larvae and mouse models [60]. They showed that the cytolysin and Fsr quorum-sensing systems were modulated by the host environmental conditions. *E*. *faecalis* strains that expressed either *gelE* or *cytolysin* induced mortality in *C. elegans* and *G. mellonella* infection models [63,95]. Whereas, combinations of *gelE* and *cytolysin* positive strains did not increase virulence significantly in both models [63,95]. This non-significant effect in the presence of both virulence traits might reflect either an antagonistic interaction or a saturation effect [63]. Gelatinase contributes to pathogenesis by triggering the proteolytic degradation of a wide range of host substrates [47,51], and is also implicated in the degradation of sex pheromone-related and other biologically active peptides [44]. In particular, the in vitro antagonistic effect of gelatinase significantly reduced the cytolysin activity of autoinducer CylL_S_ [95]. However, the combined effect of gelatinase and cytolysin in vivo could be more severe [39]. Moreover, the underlying mechanism is important and should be explored further in vivo to devise therapeutic interventions against cytolysin virulence. Overall, after autoinduction of the cytolysin operon in the presence of a target cell membrane, cytolysin is involved in hemolysis, bacterial killing, endophthalmitis, endocarditis, and other virulence traits.

## 4. Luxs System

Many bacterial species, including *E*. *faecalis*, contain conserved *luxS* homologs, which produce the LuxS enzyme. The Pfs enzyme converts *S*-adenosylhomocysteine into *S*-ribosylhomocysteine and adenine, after which LuxS cleaves its substrate, *S*-ribosylhomocysteine, into homocysteine and 4,5-dihydroxy-2,3-pentanedione (DPD), which is the precursor of AI-2 [27]. The DPD molecules react with water and undergo cyclization to form AI-2 [96]. The synthesis of AI-2 has highlighted the potential role of the LuxS system in inter-species communication among in *luxS* containing bacteria at high cell density [97,98]. The LuxS system has been well studied in the Gram-negative, marine γ-proteobacterium *Vibrio harveyi* [27]. In *V*. *harveyi*, AI-2 is sensed by a periplasmic binding protein, LuxP [99]; however, bacteria lacking LuxP also respond to AI-2. Other receptors, such as LsrB (in *Bacillus cereus*, *Escherichia coli*, and *Salmonella enterica*) and RbsB (in *Haemophilus influenza*) have also been identified as potential receptors for AI-2 (reviewed in [100]). However, in most Gram-positive bacteria, including *E*. *faecalis*, AI-2 receptors have not yet been identified.

In *E*. *faecalis*, like other Gram-positive and Gram-negative bacteria, the LuxS enzyme is involved in the transcriptional regulation of a series of genes, including those involved in ATP generation, translation, cell wall/membrane biogenesis, and nucleotide transport and metabolism [10]. However, the mechanisms that induce the deregulation of genes implicated in these pathways, remain unclear. In addition, the LuxS system in *E*. *faecalis* also plays a role in biofilm formation in vitro [10,101]. It has been suggested that the deletion of *luxS* increases biofilm formation by *E*. *faecalis* and also increases bacterial cell-surface hydrophobicity, which suggest its role in biofilm formation [101]. The addition of exogenous AI-2 to a *luxS* mutant could solve this problem of whether biofilm formation is LuxS-dependent or metabolic, because *S*-adenosylhomocysteine hydrolase converts *S*-adenosylhomocysteine to homocysteine directly and thereby restores and maintains the methionine cycle, without the formation of the DPD [102]. The role of the AI-2 signaling molecule in biofilm formation and fratricide has also been reported in *Streptococcus pneumoniae* strains, which are closely related to the enterococci [103]. Therefore, the links among biofilm development, metabolic functions, fratricide, and the lack of known receptors for AI-2 pose the question of whether AI-2 signaling in *E*. *faecalis* has any role in intercellular communication. Based on the results of previous studies, it remains unclear whether in *E*. *faecalis*, the LuxS system only represents a general metabolic function or plays a role in quorum-sensing. The intercellular communication mechanism, overall significance in biofilm development, and the metabolic functions in *E*. *faecalis* are not well understood and should be investigated further.

## 5. Therapeutic Approaches

**Figure 3:**
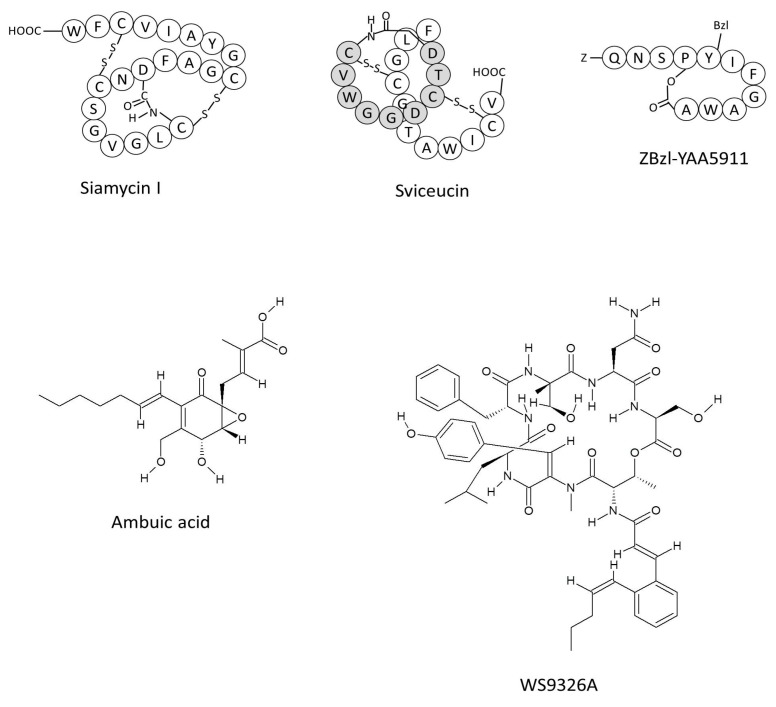
Structures of Fsr quorum-sensing system inhibitors: Siamycin I (IC_50_ approximately 100 nM) extracted from soil *Streptomyces sp* [109]; Sviceucin obtained from *Streptomyces sviceus*, (1 and 10 µM concentrations inhibited 50–70% and >90% of gelatinase production, respectively) [111]; ZBzl-YAA5911 is a synthetic inhibitor with an IC_50_ of 26.2 nM [113]; Ambuic acid (IC_50_ approximately 10 µM), extracted from fungal secondary metabolites [112]; and WS9326A (IC_50_ approximately 19 µM), extracted from a culture of actinomycetes [114].

Enterococci have exhibited resistance to almost every antibiotic used against their infections [3]. Therefore, an alternative strategy, such as quorum-sensing disruption, is one of the most exciting areas in research into multidrug-resistant bacteria [104]. Interestingly, it has been reported that quorum-sensing inhibitors might enhance host immunity by rendering the pathogen avirulent, thus making them incapable of colonizing the host [105]. Initiatives have already been taken to target the Fsr and cytolysin quorum-sensing systems to develop anti-enterococcal therapeutic drugs [82,106,107,108] (Figure 3). Actinomycete secondary metabolites, such as siamycin I, inhibited FsrC of the Fsr system and stops biofilm formation at sublethal concentrations [109]. Later, Ma et al. showed that siamycin I inhibits autophosphorylation of the histidine kinase (FsrC) [110]. Similarly, sviceucin, a member of the type I lasso peptide family, inhibited the gelatinase activity at micromolar concentrations and possibly interacts with FsrC; however, this remains to be confirmed in detail [111]. Another promising study found that ambuic acid, a fungal secondary metabolite, inhibited the proteolytic modification of FsrD by binding to FsrB [112].

Continuous progress in the field of high-throughput screening has identified new quorum-sensing inhibitors. Two compounds, Y67-1 and Y67-2, were identified by high throughput screening analyses of actinomycetes culture extracts. These compounds were further characterized and evaluated as WS9326A and WS9326B, respectively, both of which are receptor antagonists of FsrC and lead to the inhibition of Fsr activity [114]. High concentrations of sodium chloride (≥3%) have also been reported to repress the *fsr* operon, thus abolishing biofilm formation [115,116]. Such inhibition might be caused by the activation of AtlA, which also contributes to biofilm development [43]. However, the precise mechanism by which sodium chloride inhibits gelatinase and GBAP production remains unclear.

In addition to natural inhibitors, synthetic inhibitors have also sparked increasing interest because of their specific antagonistic activity against the corresponding template peptides. Nakayama et al. synthesized a GBAP analog (ZBzl-YAA5911) and assessed its inhibition efficacy systematically [113]. Their results demonstrated that ZBzl-YAA5911 significantly reduced *E*. *faecalis* counts in endophthalmitis in aphakic rabbit eyes [113]. These findings identified ZBzl-YAA5911 as a promising inhibitor for the therapeutic development to cure endophthalmitis. Besides the Fsr system inhibitors, LuxS and cytolysin synthetic analogs are also equally important. Interestingly, Piras et al. have reported that the LuxS enzyme is upregulated in multidrug-resistant bacteria [9]. This finding indicates that the suppression of quorum-sensing regulatory genes may give new insights into the control of multi-antibiotic resistant pathogens. Similarly, the molecular structure of cytolysin synthetase could permit the design of strong binding inhibitors to prevent modification of the toxin subunits. The structure of CylI is also important and might provide information regarding the interaction sites of synthetic inhibitors of cytolysin, and the immunity mechanism of *E*. *faecalis* at the molecular level.

## 6. Perspectives

To date, only two approaches, screening of natural and synthetic compounds, have been used to control the quorum-sensing mechanism in *E*. *faecalis*. These inhibitors (Figure 3) have been shown to be effective against the Fsr system. However, in vivo studies are important to understand the immune response, stability, toxicity, and metabolic pathways of quorum-sensing inhibitors. For example, siamycin I inhibits the growth of several Gram-positive bacteria and is involved in ATP-dependent enzyme activities [110]. Therefore, quorum-sensing inhibitors might also target other pathways or the normal microbiota [117]. Indeed, certain unknown factors might influence the activity of quorum-sensing inhibitors. However, the assessment of inhibitor activities in vivo, involving animal models of the disease, are very important to study immune response, as well as its detrimental effects. Ultimately, medical trials to check their effectiveness in humans are required. Furthermore, when developing new therapeutics against pathogenic *E*. *faecalis*, how commensal *E*. *faecalis*, which also releases pheromone-peptide cOB1 that is capable of killing the pathogenic *E*. *faecalis* in the gastrointestinal tract, will be spared should be taken into account [118]. Therefore, transcriptomic and proteomic analyses of quorum-sensing inhibitors seems an elegant way to determine the mechanisms used by different inhibitors with respect to virulence and biofilm formation in the pathogenic bacteria [119,120,121].

It is also noteworthy that the quorum-sensing inhibitors might also be helpful to control virulence factors. The emergence of vancomycin-resistant *E*. *faecalis*, Fsr, and cytolysin quorum-sensing systems (the LuxS system remains under debate) pose a significant medical challenge. To realize a better therapeutic effect, there is a critical need to develop quorum-sensing inhibitors [104]. The three-dimensional structures of quorum-sensing regulating proteins offer molecular-level insights into strategies to treat *E*. *faecalis* quorum-sensing related virulence [80,81,84,122]. Furthermore, in vivo studies are needed to understand the disruption of the quorum-sensing mechanism by interfering with autoinducers during the course of infection in the host. Certainly, *E*. *faecalis* is likely to evade the host immune response by releasing quorum-sensing-related virulence factors [69].

## 7. Conclusions

Important virulence factors of *E*. *faecalis* are regulated by quorum-sensing circuits and pose a significant medical and environmental threat. For instance, *E*. *faecalis* is associated with different infections, including endocarditis, endophthalmitis, ulcerative colitis, and peritonitis. It is clear that the quorum-sensing regulatory proteins gelatinase, serine protease, enterocin O16, and cytolysin are key contributors to the pathogenesis of *E*. *faecalis* in several infection models. Thus, targeting *E*. *faecalis* quorum-sensing systems by focusing specifically on the autoinducers (GBAP and CylL_S_) and their receptors might lead to design of effective drugs against *E*. *faecalis* infections. Autoinducer-antagonists are expected to interact with specific receptors and do not exert selective pressure like antibiotics. This could be a promising strategy, in terms of specificity and lack of interference with the normal host flora. However, it is not possible to exclude the interference of autoinducer antagonists with the normal host flora.

Identification of natural products using high-throughput screening procedures and modern in silico techniques, such as molecular dynamics, has opened new horizons for future drug discovery. Furthermore, in vivo studies involving synthetic and natural inhibitors are needed to determine their effect on *E*. *faecalis* pathogenesis. The in vivo efficacy studies of the quorum-sensing inhibitors, broad scale clinical trials, and the analyses of the resulting positive as well as negative aspects, remain to be performed. However, various anti-virulence approaches and their possible interference with intercellular communication should be explored, as a feasible strategy for novel drug development.

## Figures and Tables

**Table 1:** The significant contributions of the *E*. *faecalis* quorum-sensing systems to virulence against humans and model organisms.

Associated Disease	Host	Virulence Factors	Observed Activities	References
Endocarditis	Human	Fsr system	A higher prevalence of the *fsr* locus in the endocarditis isolates (100%) compared with fecal isolates (53%)	[57]
^1^ IBD	Human	Gelatinase	The expression of *gelE* gene was significantly higher in the IBD patients compared with that in the controls	[58]
IBD	Mice	Gelatinase	The *gelE* positive *E*. *faecalis* strain induced a significantly higher colitis and ileitis in the mice compared with that of the *gelE* mutant strains	[51]
Peritonitis	Mice	Cytolysin and gelatinase	Adding ^2^ SRFE to the inoculum considerably lowered the LD_50_ for *E*. *faecalis* OG1RF	[59]
Systemic infection	Mice and *G. mellonella*	Cytolysin and gelatinase	Injections of a *gelE* positive strain cause death in the *G*. *mellonella* larvae within 8 h and over 2 days in mice. Meanwhile, cytolysin was highly expressed in heart and spleen of mice	[60]
Ulcerative colitis	Mice	Gelatinase	Gelatinase can regulate intestinal permeability through ^3^ PAR2	[50]
Endophthalmitis	Rabbit	Fsr system	An *fsrB* positive strain reduced the B-wave amplitude significantly compared with an *fsrB* negative strain	[61]
Endophthalmitis	Rabbit	Gelatinase and serine protease	100 CFU/mL of *E. faecalis* OG1RF caused significant loss of retinal function after 24 h compared with *fsrB* mutant strains	[62]
Endocarditis	Rabbit	Gelatinase	A *gelE* positive phenotype increased bacterial burden in the heart tissues	[48]
Persistent infection	*C. elegans*	Fsr system and cytolysin	Feeding on lawns containing *E. faecalis* (*cytolysin* and *fsrB* positive) caused a lethal infection	[39]
Persistent infection	*C*. *elegans*	Cytolysin and gelatinase	Ingestion of different strains of *E*. *faecalis* having the *fsr* locus and *cytolysin* operon significantly increased its pathogenicity	[63]
Aerial tissue damage	*A*. *thaliana*	Fsr system	Parental strain OG1RF caused mortality after 7 days post-inoculation while *fsrB* and *sprE* mutant strains significantly attenuated virulence	[64]
Systemic infection	*D*. *melanogaster*	Gelatinase, serine protease, and enterocin O16	*gelE*, *sprE*, and *ef1097* mutant strains attenuated virulence significantly compared with the V583 parental strain	[30]

^1^ Inflammatory Bowel Diseases (IBD); ^2^ Sterile Rat Fecal Extracts (SRFE); ^3^ Protease-Activated Receptor 2 (PAR2).

## Abbreviations

2D-PAGE	Two-dimensional polyacrylamide gel electrophoresis
Agr	Accessory gene regulator
AI-2	Autoinducer 2
AtlA	*N*-acetylglucosaminidase
CFU	Colony forming units
DPD	4,5-dihydroxy-2,3-pentanedione
Fsr	Faecalis system regulator
GBAP	Gelatinase biosynthesis activating pheromone
IBD	Inflammatory bowel diseases
PAR2	Protease-activated receptor 2
SRFE	Sterile rat fecal extracts
